# Pellino-1 Regulates the Responses of the Airway to Viral Infection

**DOI:** 10.3389/fcimb.2020.00456

**Published:** 2020-08-31

**Authors:** Elizabeth K. Marsh, Elizabeth C. Prestwich, Lynne Williams, Amber R. Hart, Clare F. Muir, Lisa C. Parker, Marnix R. Jonker, Irene H. Heijink, Wim Timens, Mark Fife, Tracy Hussell, Marc B. Hershenson, J. Kelley Bentley, Shao-Cong Sun, Ben S. Barksby, Lee A. Borthwick, James P. Stewart, Ian Sabroe, David H. Dockrell, Helen M. Marriott

**Affiliations:** ^1^Department of Infection, Immunity and Cardiovascular Disease, Faculty of Medicine, Dentistry and Health, University of Sheffield, Sheffield, United Kingdom; ^2^Human Sciences Research Centre, College of Life and Natural Sciences, University of Derby, Derby, United Kingdom; ^3^Department of Pathology and Medical Biology, University of Groningen, University Medical Centre Groningen, Groningen, Netherlands; ^4^Manchester Collaborative Centre for Inflammation Research, Core Technology Facility, University of Manchester, Manchester, United Kingdom; ^5^Department of Pediatrics and Communicable Diseases, University of Michigan Medical School, Ann Arbor, MI, United States; ^6^Department of Immunology, University of Texas MD Anderson Cancer Center, Houston, TX, United States; ^7^Newcastle Fibrosis Research Group, Institute of Cellular Medicine, Newcastle University, Newcastle upon Tyne, United Kingdom; ^8^Department of Infection Biology, University of Liverpool, Liverpool, United Kingdom; ^9^MRC/UoE Centre for Inflammation Research, Queen's Medical Research Institute, University of Edinburgh, Edinburgh, United Kingdom

**Keywords:** Pellino-1, influenza A virus, rhinovirus, COPD, asthma, airway epithelia, inflammation

## Abstract

Exposure to respiratory pathogens is a leading cause of exacerbations of airway diseases such as asthma and chronic obstructive pulmonary disease (COPD). Pellino-1 is an E3 ubiquitin ligase known to regulate virally-induced inflammation. We wished to determine the role of Pellino-1 in the host response to respiratory viruses in health and disease. Pellino-1 expression was examined in bronchial sections from patients with GOLD stage two COPD and healthy controls. Primary bronchial epithelial cells (PBECs) in which Pellino-1 expression had been knocked down were extracellularly challenged with the TLR3 agonist poly(I:C). C57BL/6 *Peli1*^−/−^ mice and wild type littermates were subjected to intranasal infection with clinically-relevant respiratory viruses: rhinovirus (RV1B) and influenza A. We found that Pellino-1 is expressed in the airways of normal subjects and those with COPD, and that Pellino-1 regulates TLR3 signaling and responses to airways viruses. In particular we observed that knockout of Pellino-1 in the murine lung resulted in increased production of proinflammatory cytokines IL-6 and TNFα upon viral infection, accompanied by enhanced recruitment of immune cells to the airways, without any change in viral replication. Pellino-1 therefore regulates inflammatory airway responses without altering replication of respiratory viruses.

## Introduction

Exacerbations of airways diseases remain a major challenge for respiratory medicine. Viruses are major causes of acute exacerbations in both asthma and COPD, with human rhinovirus being the most commonly associated virus (Sethi and Murphy, 2008; Leigh and Proud, 2015), and influenza A associated with more serious COPD exacerbations requiring hospitalization (Sethi and Murphy, 2008). New molecular targets of virus-induced inflammation would have enormous therapeutic potential by reducing excessive and inappropriate inflammation without preventing host defense functions. Based on previous work from our group (Bennett et al., 2012), we examined the role of Pellino-1 in the control of viral-induced airway inflammation.

Signaling pathways are commonly controlled by phosphorylation, but are also regulated by the pathway of ubiquitination (Moynagh, 2014). Pellino-1 is an E3 ligase whose knockdown or inhibition resulted in decreased responses to IL-1 (Jiang et al., 2003). Mice deficient in Pellino-1 showed reduced signs of sepsis following systemic stimuli activating the innate immune receptors TLR3 and TLR4 (Chang et al., 2009). We previously showed that Pellino-1 knockdown in human airway epithelial cells reduced inflammatory responses to rhinoviral infection, without altering rhinoviral replication (Bennett et al., 2012), and a similar decrease in proinflammatory cytokines has been observed upon Pellino-1 silencing in human myometrial cells after TNF and TLR stimulation (Lim et al., 2018). Other studies showed additional roles for Pellino-1 in limiting development of autoimmunity; aged Pellino-1-deficient mice develop an autoimmune phenotype with excess T cell activation compared to aged wild type mice (Chang et al., 2011). Pellino-1 expression may also be increased in asthma and COPD (Baines et al., 2011, 2013).

Pellino-1 is therefore widely expressed with roles in the regulation of TLR3 and TLR4 signaling and autoimmunity. However, the role of Pellino-1 in the responses to respiratory viruses *in vivo* has not been explored. We therefore addressed the knowledge gap in this work. Our new data shows Pellino-1 acts as a negative regulator of inflammatory airway responses to influenza and rhinovirus, a role dissociated from control of viral replication.

## Materials and Methods

### Cell Culture, Transfection, and Stimulation

Primary Bronchial Epithelial Cells (PBECs) isolated from healthy human volunteers (*n* = 4) were from PromoCell and maintained as described previously (Bennett et al., 2012). Expression of Pellino-1 was knocked down in epithelial cells using Dharmacon ON-TARGET plus SMARTpool™ short interfering RNA (siRNA) system and Lipofectamine 2000 (Bennett et al., 2012); successful knock down was confirmed by immunoblot for Pellino-1/2 (mouse monoclonal antibody, clone F-7, Santa Cruz) each time (Supplementary Figure 1). Cells were equilibrated in basal media for a minimum of 4 h before stimulation, and harvested for RNA or protein analysis, and cell-free supernatants taken at the time points indicated. Poly(I:C) from Invivogen (San Diego) was used at 50 μg/ml.

### Patients and Immunohistochemistry

Lung tissue was taken from lungs of seven patients with COPD characterized as GOLD stage two, seven age-matched current smokers, and eight age-matched non-smokers. Tissue was derived from leftover lung material of tumor resection surgery, which was always taken far distant from the tumor and checked for abnormalities by experienced pathologists. Subject exclusion criteria were the diagnosis of asthma, indications of lung infection or other lung pathologies. The study protocol was consistent with the Research Code of the University Medical Center Groningen https://www.rug.nl/umcg/research/documents/research-code-info-umcg-nl.pdf and National Ethical and Professional Guidelines (“Code of conduct; Dutch Federation of Biomedical Scientific Societies,” http://www.federa.org). Sections were embedded in paraffin and stained for Pellino-1/2 (mouse monoclonal antibody, clone F-7, Santa Cruz) following a Tris-EDTA antigen retrieval. Specific staining was checked when setting up the assay with a non-specific mouse isotype control, which was negative. A secondary antibody control was run with the staining (primary antibody replaced with PBS), which also was negative. We have previously shown that Pellino-2 is not expressed in the human airway epithelium (Bennett et al., 2012), therefore specific staining to Pellino-1 was observed in the airway epithelial cells. These cells were analyzed specifically, rather than the entire lung section; the epithelium was highlighted using the Aperio software in multiple airways within each section. Because the number of airways were different for each patient, the intensity was normalized for area. Positive area-corrected-pixels were then divided by negative area-corrected-pixels to give a ratio of positive to negative pixels within the lung epithelium for each patient. The epithelial airways staining was scored using Leica Imagescope Aperio software to give a computerized quantitative analysis of ratio of intensity corrected for area.

### Mice

Specific pathogen-free age- and sex-matched wild type and *Peli1*^−/−^ mice (Chang et al., 2009) were maintained in-house with food and water provided *ad libitum*. All work involving animals was performed in accordance with the Animal (Scientific Procedures) Act 1986 and was approved by the animal welfare and ethical review bodies at the University of Sheffield and the University of Manchester. Work was carried out under procedure project licenses 40/3726 (Sheffield) and 70/7460 (Manchester), and met ARRIVE guidelines. All animals were checked prior to the start of experiments by competent personal licensees (PIL), granted under Animal (Scientific Procedures) Act 1986; all animals were deemed to be fit and well before the start of experiments. The breeding colony used to supply these experiments was maintained as a het × het closed colony, in house, with the genotype of individual animals confirmed by PCR prior to use. Before each experiment, animals were age- and sex-matched and randomly distributed amongst the groups, and treatments administered by a blinded PIL technician. All procedures were performed first thing in the morning after the 12 h dark cycle. *N* numbers in figure legends correspond to single animals. Power calculations based on representative results of alveolar macrophage innate responses measured within our established pulmonary infection models with bacteria and viruses were used to inform group sizes (Dockrell et al., 2003).

Where indicated, bone marrow derived progenitor cells were differentiated into macrophages over 14 days in the presence of L929-conditioned media (Chan et al., 1999), before stimulating with 25 μg/ml poly(I:C).

### ELISA

Cell-free fluids were collected and stored at −80°C. ELISAs were performed using antibodies from R&D Systems. Mouse IgM was quantified using Mouse IgM ELISA Quantitation Set (Bethyl Laboratories). Total protein was assessed by Bradford assay (BioRad).

### Systemic Sepsis Model

Wild type or litter mate controls, housed in the same cages, were intraperitoneally injected with poly(I:C) (0.5 μg/g body weight) plus D-Galactosamine (0.7 mg/g) as described (Chang et al., 2009), or an equal volume of PBS, and monitored for the first 3 h following injection; animals were sacrificed at the time points indicated by terminal anesthesia. Blood was collected from the caudal vena cava.

### Pulmonary Stimulation and Infection

Mice were anesthetized using 3–5% gaseous isoflurane. Stimuli were administered intranasally (i.n.), using 50 μl PBS solutions containing 100 μg poly(I:C), 1 × 10^6^ PFU RV1B, or 5 × 10^3^ PFU influenza A virus X31 (H3N2). Controls for the poly(I:C) and IAV challenge were PBS, and for the RV challenge was sham infected cell lysate, processed in a manner identical to the RV. Mice were placed in a warmed cage until they were moving freely after anesthesia, and allowed to recover with access to food (supplemented with forage mix), and water, and weighed and monitored daily for signs of respiratory distress.

### Analysis of Murine Airways

Mice were euthanized by a terminal dose of intraperitoneal anesthetic (sodium pentobarbitone 20%); death was confirmed by exsanguination via the femoral artery. BAL was performed using 3 × 1 ml aliquots of ice-cold PBS, and cellular composition analyzed as described (Dockrell et al., 2003). Blood was harvested from the caudal vena cava. Cell-free bronchoalveolar lavage fluid (BALF), serum, lung, and supernatant samples were stored at −80°C. Cytokines were quantified using the Mouse Th1/Th2/Th17 cytokine kit, or KC, IL-6, TNFα, CCL2, and CCL5 Flex Sets for cytometric bead array (BD Biosciences).

### Quantitative PCR

RNA was prepared from cell lysates and homogenized murine lungs by TRI Reagent or acid-Phenol:Cholorform:IAA 125:24:1 pH 4.5 extraction (Ambion), respectively. cDNA was prepared using high-capacity cDNA reverse transcriptase kit (Applied Biosystems), genomic DNA removed by DNA-*free*™, and qPCR performed using GoTaq® master mix (Promega, Madison, US) and primer-probe sets from Applied Biosystems. Cell culture samples were quantified against a plasmid standard curve containing known copy numbers of target genes; murine samples were analyzed using the relative quantification (2^−ΔΔCt^) method to 18sRNA.

Probes used were GAPDH Hs00182082_m1; IFNβ Hs01077958_s1; murine CXCL10 Mm00445235_m1; murine IFNβ Mm00439552_s1; murine IL-6 Mm00446190_m1; murine Rn18s Rn45s Mm04277571_s1;

RV1B F 5′-GTGAAGAGCCSCRTGTGCT-3′,
R 5′-GCTSCAGGGTTAAGGTTAGCC-3′,
probe 5′-[6FAM]TGAGTCCTCCGGCCCCTGAATG[TAM]-3′;
X31 F 5′-CATCCTGTTGTATATGAGGCCCAT-3′,
R 5′-GGACTGCAGCGTAGACGCTT-3′,
probe 5′-[6FAM]CTCAGTTATTCTGCTGGTGCACTTGCCA[TAM]-3′.

### Histology

The inferior lobe of the murine lung was fixed in 10% formalin, transferred into 70% ethanol, then embedded in paraffin; 4 μM sections were prepared and stained with H&E, and evaluated on an Olympus BH-2 microscope.

### Precision Cut Lung Slice (PCLS) Model

Explanted lung tissue was placed in isotonic saline at 4°C and transported for processing within 24 h. Lung tissue was warmed to 37°C for 60 min in HBSS+ and then inflated with 3 ml of 3% low melting point agarose by cannulation of the trachea. Inflated lung tissue was incubated at 4°C on ice to allow the agarose to set and cored using an 8 mm skin biopsy punch. Three cores were placed upright in a metal mold with additional 3% low melting point agarose and incubated at 4°C on ice for 30 min to allow the agarose to set. Tissue cores embedded in agarose were super-glued to the vibratome mounting stage, submersed in ice-cooled HBSS+ in the media chamber and then cut using a Leica VT1200S vibrating blade microtome (Leica Biosystems) at a speed 0.3 mm/s, amplitude 2 mm and thickness (step size) of 450 μm. PCLS were transferred to 8 μm pore transwell-inserts in 24 well plates and cultured in 500 μl/well of Small Airway Epithelial Cell Basal Medium and supplements (PromoCell) and antibiotics. Media was refreshed every 24 h. PCLS were rested for 48 h prior to exogenous treatment with poly(I:C) or LPS for 24 h. Cytokines in cell culture supernatants from PCLS were quantified using Meso Scale Discovery electrochemiluminescence detection kits according to manufacturer's instructions.

### Flow Cytometry

BALF was collected before lungs were excised. Lungs were chopped and digested with Liberase™ (Roche) and DNAse and strained. Red cells were lysed with HyBri-Max™ (Sigma), and protein secretion blocked with Brefeldin A. Cells were stained with Live/Dead Zombie UV Fixable Viability Kit (Biolegend) and Fc block CD16/CD32 monoclonal antibody (93; eBioscience). Antibodies used were FITC anti-mouse CD8a [53-6.7] (Biolegend), PerCP/Cy5.5 anti-mouse CD4 [GK1.5] (Biolegend), TCR gamma/delta Monoclonal Antibody [eBioGL3 (GL-3, GL3)] APC (eBioscience), APC/Cy7 anti-mouse CD19 [6D5] (Biolegend), CD3 Monoclonal Antibody (OKT3) eFluor 450 (eBioscience), Brilliant Violet 510 anti-mouse CD45 [30-F11] (Biolegend), Brilliant Violet 650 anti-mouse NK-1.1 [PK13b] (Biolegend), Anti-Mouse Ly-6G (Gr-1) FITC (eBioscience), Anti-Mouse CD11b PerCP-Cyanine5.5 (eBioscience), Alexa Fluor® 700 anti-mouse I-A/I-E Antibody (Biolegend), Anti-mouse Ly-6C eFluor450 [HK1.4] (Biolegend), Brilliant Violet 510 anti-mouse CD45 [30-F11] (Biolegend), Brilliant Violet 650™ anti-mouse CD11c [N450] (Biolegend), and PE/Cy7 anti-mouse CD64 (FcγRI) (Biolegend). Intracellular staining of IFNγ-PE [PE anti-mouse IFN-γ [XMG1.2] (Biolegend)] and IL-17 [APC anti-mouse IL-17A [TC11-18H10.1] (Biolegend)] was performed after cell permeabilisation. Electronic compensation was performed with UltraComp eBeads (eBiosciences). Data were acquired on a BD LSR Fortessa, and analyzed using FlowJo (TreeStar Inc). Total numbers of cells were calculated using CountBright™ Beads.

### Statistical Analysis

Data generated typically required multiple comparisons and were therefore presented as mean ± SEM and analyzed by two-way ANOVA with post-testing. Where *n* was <6 the Shapiro-Wilk normality test was used to confirm that significant differences were in datasets where no deviation from normality was detected (not shown).

## Results

**Figure 1:**
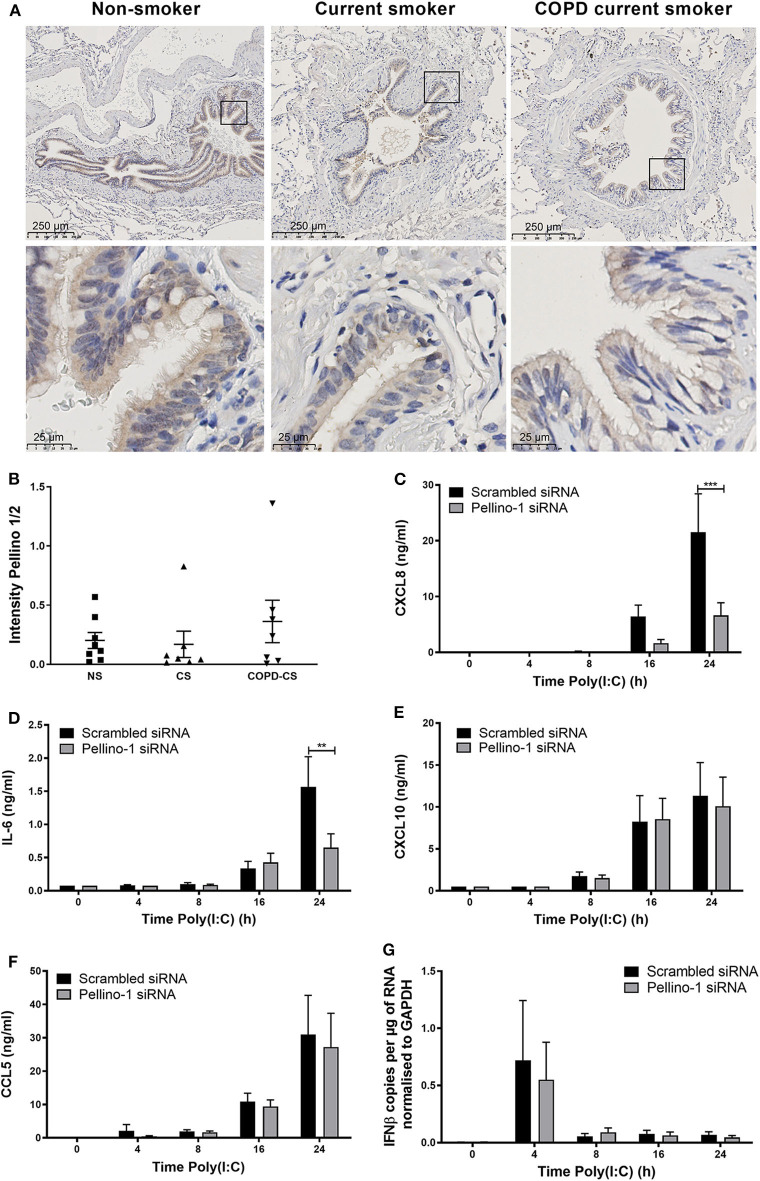
Pellino-1 is expressed in the airway epithelium and regulates the proinflammatory epithelial response to TLR3 activation. Lung tissue sections from COPD patients with GOLD stage II (*n* = 7), age-matched smoking controls (*n* = 7), and age-matched non-smokers (*n* = 8) were stained for Pellino-1/2. Representative images are shown **(A)**, and staining intensity quantified **(B)** with differences between groups analyzed by Mann-Whitney *U*-test; no significance was found. PBECs were transfected with 100 nM siRNA targeting Pellino-1 or scrambled (control) for 24 h, prior to treatment with poly(I:C) (50 μg/ml) over 24 h. Cell lysates and supernatants were collected and CXCL8 **(C)**, IL-6 **(D)**, CXCL10 **(E)**, and CCL5 **(F)** release measured by ELISA. IFNβ expression was measured by qRT-PCR **(G)**. Data shown are mean ± SEM, *n* = 4. Significance between siRNA treatments is indicated by **p < 0.01 and ***p < 0.001 as measured by two-way ANOVA with Sidak's post-test.

Although Pellino-1 expression has been described in human tissues *in vitro*, protein expression patterns in the human lung *in vivo* are not known. We studied tissue samples from patients with COPD, a chronic inflammatory disease characterized by frequent viral exacerbations with inflammatory sequelae in the airway. We observed that Pellino-1 is mainly expressed in the airway epithelium with similar levels of expression in healthy controls, smokers, and patients with GOLD Stage two COPD, rendering it a potentially viable therapeutic target (Figures 1A,B). Patient characteristics are shown in Supplementary Table 1.

We next confirmed that Pellino-1 is a regulator of human airway epithelial cell signaling, using primary human epithelial cells. Consistent with our previous results (Bennett et al., 2012), Pellino-1 knockdown by siRNA reduced the induction of proinflammatory CXCL8 and IL-6 release upon TLR3 activation at 24 h post-challenge (Figures 1C,D). However, markers of the antiviral responses such as CXCL10 and CCL5 release, and IFNβ expression, were unchanged (Figures 1E–G). There were no significant differences observed in any of the measured cytokines at earlier time points (4–16 h).

**Figure 2:**
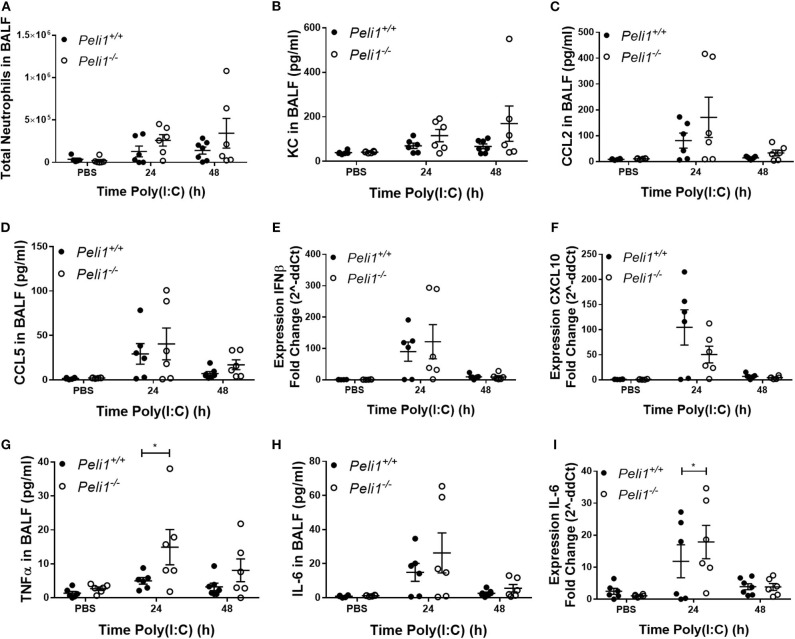
*Peli1*^−/−^ mice show increased proinflammatory responses to TLR3 activation. *Peli1*^−/−^ mice and age- and sex-matched wild type littermate controls were given a single dose of 100 μg poly(I:C) or PBS (control) i.n. under gaseous anesthesia. Animals were sacrificed and lavaged at the time points indicated, and total cell number in BALF enumerated by haemocytometer, with the number of neutrophils determined by differential cell counts calculated by microscopic analysis of cytospin preparations **(A)**. Levels of the cytokines KC **(B)**, CCL2 **(C)**, CCL5 **(D)**, TNFα **(G)**, and IL-6 **(H)** in BALF were determined by CBA. IFNβ **(E)**, CXCL10 **(F)**, and IL-6 **(I)** expression levels were determined by qRT-PCR of homogenized whole lung. Individual data points show a single mouse, and data panels show mean ± SEM; *Peli1*^+/+^ mice PBS *n* = 6, PIC 24 h *n* = 6, PIC 48 h *n* = 6; *Peli1*^−/−^ mice PBS *n* = 6, PIC 24 h *n* = 6, PIC 48 h *n* = 6. Significant differences between groups are indicated by *p < 0.05, as measured by two-way ANOVA with Sidak's post-test (statistical analysis was conducted upon dCt values for qRT-PCR data).

Loss of Pellino-1 has been shown to protect against septic shock induced by TLR3 ligands (Chang et al., 2009). We recapitulated these *in vivo* murine observations by intraperitoneal injection of poly(I:C) + D-Galactosamine and confirmed that genetic deficiency of Peli1 inhibited IL-6 and TNFα levels in serum (Supplementary Figure 2). Having confirmed this phenotype, we determined if *Peli1*^−/−^ reduced inflammatory responses to TLR3 activation in the airways. Using neutrophils as a marker of the inflammatory response elicited to intranasal instillation of poly(I:C), we found no difference in the total numbers of neutrophils recruited to the airways between wild type and knockout animals (Figure 2A), and found no reduction in KC or CCL2 levels in BALF of *Peli1*^−/−^ mice (Figures 2B,C). Levels of CCL5 and IFNβ were also preserved in these animals, although there was an isolated non-significant reduction in CXCL10 expression (Figures 2D–F). Surprisingly, and in contrast to results seen with intraperitoneal poly(I:C) administration, at 24 h post-instillation we observed higher levels of TNFα and IL-6 in the BALF of knockout animals stimulated with poly(I:C) (Figures 2G,H), and upregulated expression of IL-6 mRNA in the lung (Figure 2I), though expression of these cytokines had decreased by 48 h.

These data suggested that Pellino-1 may play a negative regulatory role to viral infection in the lung. We therefore studied the responses of *Peli1*^−/−^ animals to two viral respiratory pathogens.

**Figure 3:**
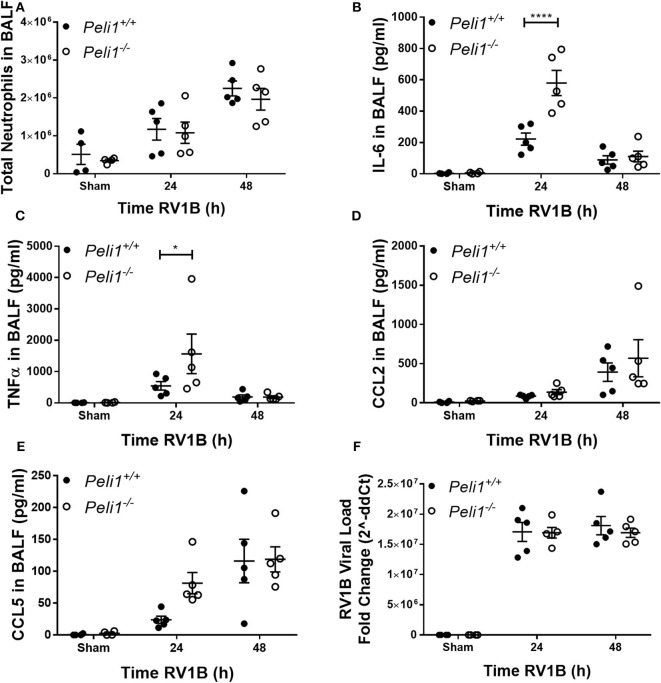
Peli1 limits proinflammatory airway responses to high-dose treatment with RV1B. *Peli1*^−/−^ mice and age- and sex-matched wild type littermate controls were given a single dose of 5 × 10^6^ PFU RV1B or sham (control; HeLa H1 cell lysate processed in a manner identical to the RV1B-infected cell lysates) i.n. under gaseous anesthesia. Animals were sacrificed and lavaged at the time points indicated, and total cell number in BALF enumerated by haemocytometer, with the number of neutrophils determined by differential cell counts calculated by microscopic analysis of cytospin preparations **(A)**. Levels of the cytokines IL-6 **(B)**, TNFα **(C)**, CCL2 **(D)**, and CCL5 **(E)** in BALF were determined by CBA. RV1B viral load **(F)** was determined by qRT-PCR of homogenized whole lung. Individual data points show a single mouse, and data panels show mean ± SEM; *Peli1*^+/+^ mice sham *n* = 4, RV1B 24 h *n* = 6, RV1B 48 h *n* = 5; *Peli1*^−/−^ mice sham *n* = 4, RV1b 24 h *n* = 5, RV1B 48 h *n* = 5. Significant differences between groups are indicated by *p < 0.05, and ****p < 0.0001, as measured by two-way ANOVA with Sidak's post-test (statistical analysis was conducted upon dCt values for qRT-PCR data).

We examined responses to intranasal rhinovirus instillation using a high-titer pure RV1B preparation (Newcomb et al., 2008). We found no difference in the number of neutrophils recruited to the airway lumen between wild type and knockout animals (Figure 3A). However, as with responses against poly(I:C), we observed higher levels of IL-6 and TNFα in the *Peli1*^−/−^ mice 24 h post-instillation (Figures 3B,C); again, these levels had decreased by 48 h. Levels of CCL2 and CCL5 were unchanged between the groups (Figures 3D,E). KC production was below the level of detection (data not shown). We found no difference in the viral load between wild type and knockout animals (Figure 3F).

**Figure 4:**
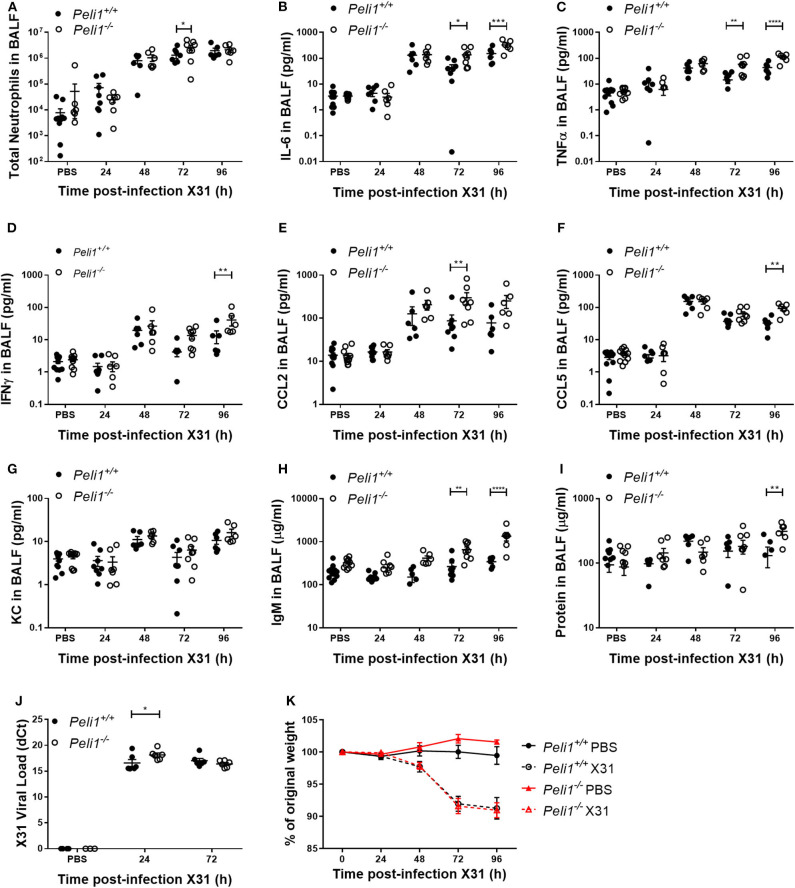
*Peli1*^−/−^ mice exhibit enhanced proinflammatory responses and airway damage to influenza A infection, without loss of viral replication. *Peli1*^−/−^ mice and age- and sex-matched wild type littermate controls were given a single dose of 5 × 10^3^ PFU influenza A X31 or PBS (control) i.n. under gaseous anesthesia. Animals were sacrificed and lavaged at the time points indicated, and total cell number in BALF enumerated by haemocytometer, with the number of neutrophils determined by differential cell counts calculated by microscopic analysis of cytospin preparations **(A)**. Levels of the cytokines IL-6 **(B)**, TNFα **(C)**, IFNγ **(D)**, CCL2 **(E)**, CCL5 **(F)**, and KC **(G)** in BALF were determined by CBA. IgM levels **(H)** in BALF were determined by ELISA. Total protein levels **(I)** in BALF were determined by Bradford assay. X31 viral load **(J)** was determined by qRT-PCR of homogenized whole lung. Animals were weighed daily, and weight loss is shown as a percentage of original weight **(K)**. Individual data points **(A–J)** show a single mouse, and data panels **(A–K)** show mean ± SEM; *Peli1*^+/+^ mice PBS *n* = 12, X31 24 h *n* = 8, X31 48 h *n* = 6, X31 72 h *n* = 8, X31 96 h *n* = 6; *Peli1*^−/−^ mice PBS *n* = 11, X31 24 h *n* = 7, X31 48 h *n* = 6, X31 72 h *n* = 8, X31 96 h *n* = 6. Significant differences between groups are indicated by *p < 0.05, **p < 0.01, ***p < 0.001, and ****p < 0.0001, as measured by two-way ANOVA with Sidak's post-test (statistical analysis was conducted upon dCt values for qRT-PCR data).

**Figure 5:**
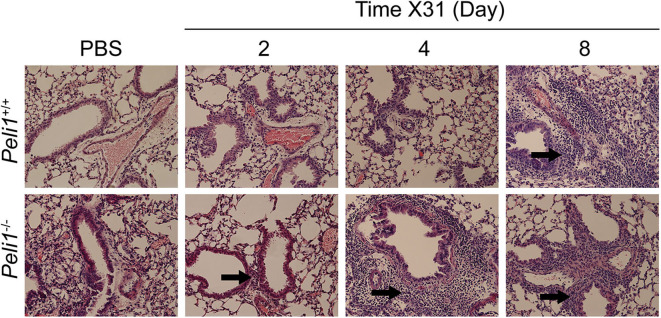
Histological examination of the murine lung with influenza A (X31) infection. *Peli1*^−/−^ mice and age- and sex-matched wild type littermate controls were given a single dose of 5 × 103 PFU influenza A X31 or PBS (control) i.n. under gaseous anesthesia. Animals were sacrificed at the time points indicated, and the inferior lobe of the lung was harvested, fixed in 10% formalin, transferred into 70% ethanol, then embedded in paraffin, and 4 μM sections prepared and stained with H&E. Sections were evaluated by a blinded board-certified veterinary pathologist (C.M.) and imaged on an Eclipse E600 microscope (Nikon) with NISElements BR software, at 20 × magnification. Areas of inflammatory cell infiltrates surround bronchioles (arrows). *Peli1*^+/+^ mice PBS *n* = 4, X31 2 day *n* = 4, X31 4 day *n* = 4, X31 8 day *n* = 4; *Peli1*^−/−^ mice PBS *n* = 4, X31 2 day *n* = 4, X31 4 day *n* = 3, X31 8 day *n* = 4.

Different respiratory viruses are individual in their modes of infection, and rhinovirus replicates poorly in the mouse. We therefore compared results with mice infected intranasally with a sub-lethal dose of influenza A X31. In this model we observed a significant increase in total neutrophils in *Peli1*^−/−^ animals at 72 h post-instillation (Figure 4A), and again saw higher levels of IL-6 and TNFα in BAL fluid at both 72 and 96 h post-infection (Figures 4B,C). IFNγ, CCL2, and CCL5 levels were also higher in knockout mice, whilst KC levels were again low in both groups (Figures 4D–G). We saw an increase in early tissue injury, with increased leakage of IgM and total protein into the BALF of *Peli1*^−/−^ animals at 96 h post-infection (Figures 4H,I), although histological examination of the lung over 8 days showed similar progressive injury to the bronchial and bronchiolar epithelium between groups (Figure 5). There was a small increase in X31 viral mRNA load at 24 h in *Peli1*^−/−^ mice when compared to wild type mice, which was not maintained at 72 h (Figure 4J). In keeping with this, we observed no difference in the animals' weight loss (Figure 4K).

**Figure 6:**
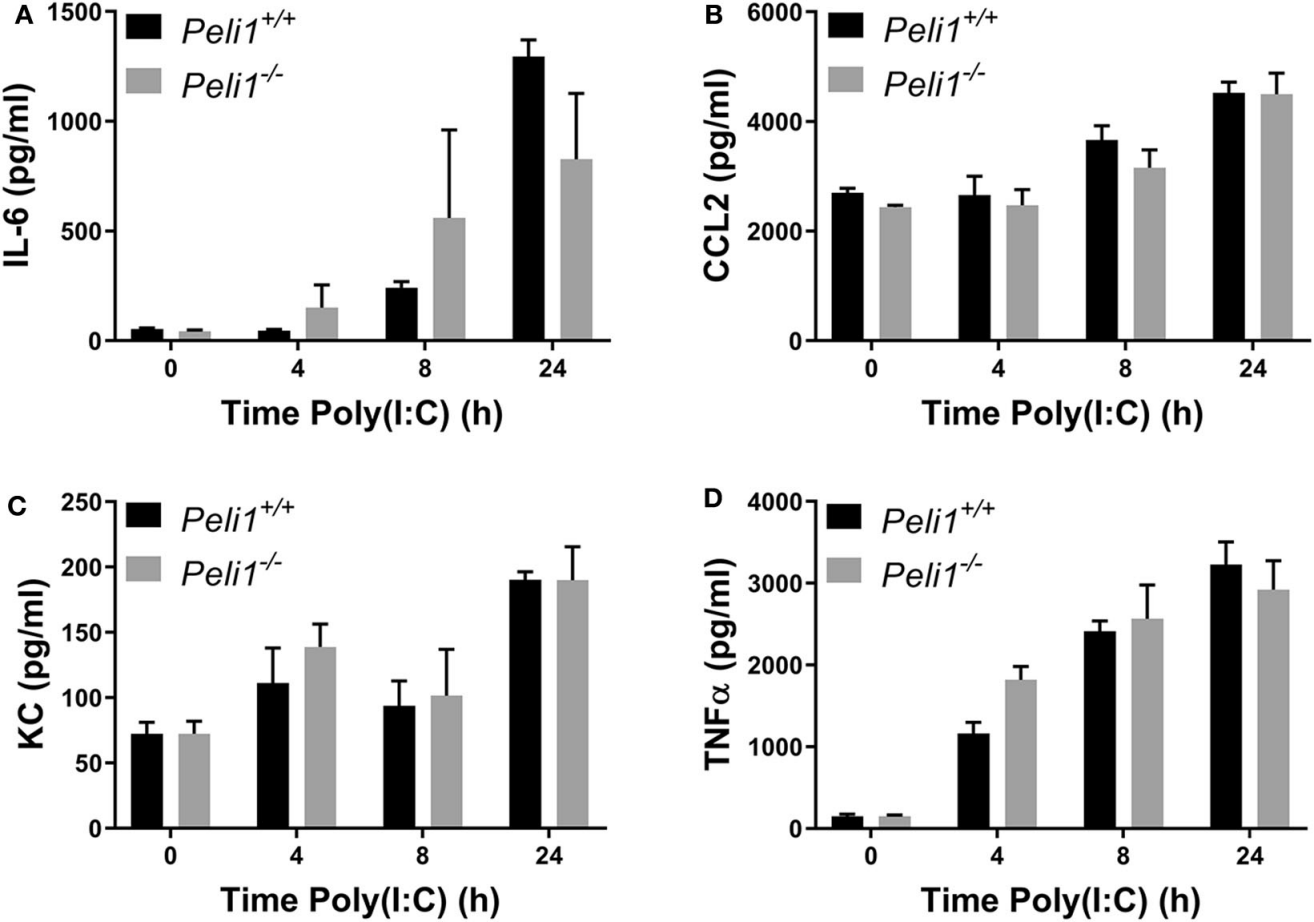
Peli1 does not regulate responses to TLR3 activation in bone marrow derived macrophages. Bone marrow derived progenitor cells from the femurs of age- and sex-matched *Peli1*^+/+^ (*n* = 4) and *Peli1*^−/−^ mice (*n* = 4) were isolated and differentiated into macrophages over 14 days in the presence of L929-conditioned media. BMDMs were stimulated with 25 μg/ml poly(I:C) across 24 h. Cell supernatants were collected and IL-6 **(A)**, CCL2 **(B)**, KC **(C)**, and TNFα **(D)** release measured by CBA. Data shown are mean ± SEM. No statistically significant differences were found.

We next examined if Pellino-1 was mediating its roles through signaling in tissue cells or leukocytes. We examined responses of bone marrow derived macrophages from age- and sex-matched wild type and *Peli1*^−/−^ mice. There were no differences in the production of IL-6, CCL2, KC, or TNFα between these BMDM populations in response to poly(I:C) (Figures 6A–D). The absence of an increased cytokine generation from BMDMs suggest that the altered response to viral stimuli was not dependent upon macrophages.

**Figure 7:**
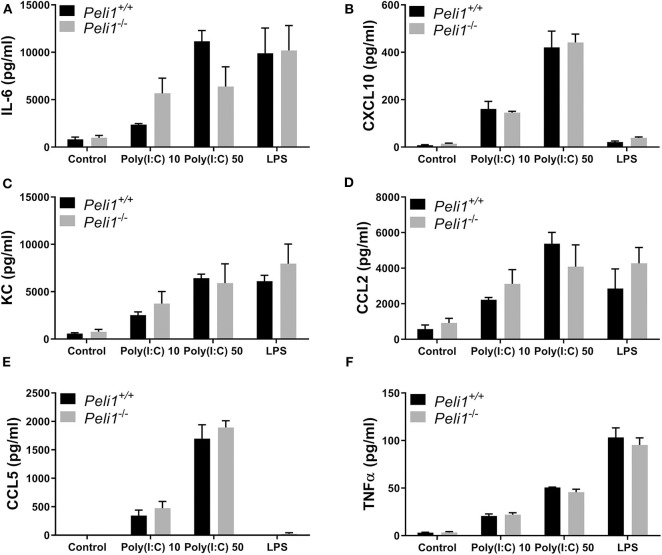
Peli1 does not regulate the response of resident immune cells to TLR3 activation in the murine lung. Explanted lung tissue from age- and sex-matched *Peli1*^+/+^ (*n* = 3) and *Peli1*^−/−^ mice (*n* = 3) was stored at 4°C in isotonic saline and transported to Newcastle University for processing. Precision cut lung sections were cultured *in vitro* and rested for 48 h prior to treatment with poly(I:C) or LPS for 24 h at the concentrations indicated. Cell culture supernatants were analyzed for IL-6 **(A)**, CXCL10 **(B)**, KC **(C)**, CCL2 **(D)**, CCL5 **(E)**, and TNFα **(F)** release using MSD electrochemiluminescence detection. Data shown are mean ± SEM. No statistically significant differences were found.

We next studied precision-cut lung slices taken from animals that had not been treated with any agonist or drug. Precision-cut lung slices are a model system that allow investigation of responses from multiple tissue cells, and stimulation of these *ex vivo* slices examines the responses of lungs without any contribution from recruited leukocytes. Lung slices from wild type and *Peli1*^−/−^ mice were stimulated following *ex vivo* culture. These data revealed no difference in the response of whole lung tissue of knockout mice vs. wild type when stimulated with poly(I:C) or LPS (Figure 7).

**Figure 8:**
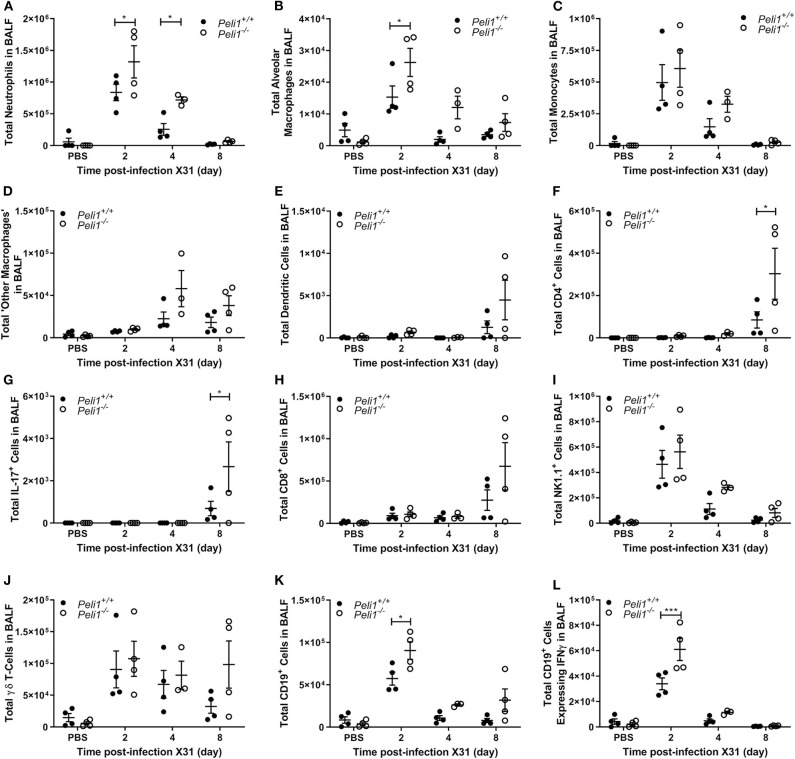
*Peli1*^−/−^ animals recruit increased numbers of immune cells to the airways following influenza A virus infection. *Peli1*^−/−^ mice and age- and sex-matched wild type littermate controls were given a single dose of 5 × 10^3^ PFU influenza A X31 or PBS (control) i.n. under gaseous anesthesia. Animals were sacrificed and lavaged at the time points indicated. Live single immune cell types were analyzed by flow cytometry **(A–L)**; gating strategies for defining subpopulations are shown in Supplementary Figure 3. Total numbers of cells were calculated using CountBright™ Beads (Invitrogen). Individual data points show a single mouse, and data panels show mean ± SEM; *Peli1*^+/+^ mice PBS *n* = 4, X31 2 day *n* = 4, X31 4 day *n* = 4, X31 8 day *n* = 4; *Peli1*^−/−^ mice PBS *n* = 4, X31 2 day *n* = 4, X31 4 day *n* = 3, X31 8 day *n* = 4. Significant differences between groups are indicated by *p < 0.05, and ***p < 0.001, as measured by two-way ANOVA with Sidak's post-test.

**Figure 9:**
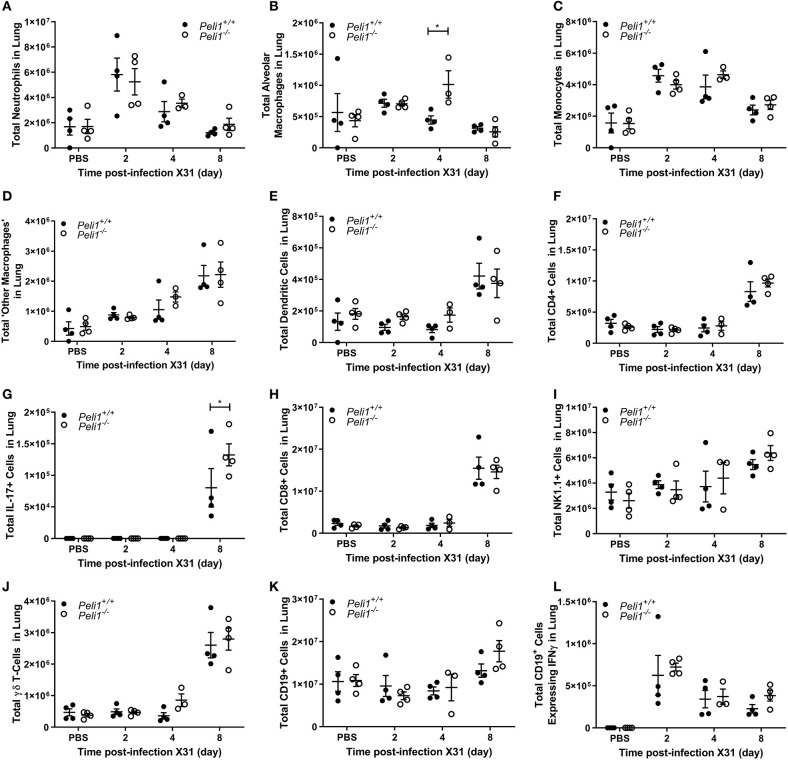
Characterization of recruited immune cells to the lung interstitium following influenza A virus infection. *Peli1*^−/−^ mice and age- and sex-matched wild type littermate controls were given a single dose of 5 × 10^3^ PFU influenza A X31 or PBS (control) i.n. under gaseous anesthesia. Animals were sacrificed at the time points indicated. Lungs were excised after lavage (data shown in Figure 8), digested, strained, and live single immune cell types were analyzed by flow cytometry **(A–L)**; gating strategies for defining subpopulations are shown in Supplementary Figure 3. Total numbers of cells were calculated using CountBright™ Beads (Invitrogen). Individual data points show a single mouse, and data panels show mean ± SEM; *Peli1*^+/+^ mice PBS *n* = 4, X31 2 day *n* = 4, X31 4 day *n* = 4, X31 8 day *n* = 4; *Peli1*^−/−^ mice PBS *n* = 4, X31 2 day *n* = 4, X31 4 day *n* = 3, X31 8 day *n* = 4. Significant differences between groups are indicated by *p < 0.05, as measured by two-way ANOVA with Sidak's post-test.

To further explore the nature of the inflammatory response to viruses and TLR3 agonists in *Peli1*^−/−^ animals, we examined the cells recruited to the airways in response to influenza infection over 8 days by flow cytometry. Mice were infected with influenza A (i.n.) and the immune infiltrate in lung and BAL characterized. Consistent with observations shown in Figure 4, we observed a significant increase in the total numbers of immune cells in the BAL fluid of *Peli1*^−/−^ animals (Figure 8), with a significant increase in neutrophils at day 2 and 4 post-infection, and alveolar macrophages at day 2 (Figures 8A,B). Knockout animals also showed increased recruitment of CD4^+^ T cells, Th17 (CD4^+^IL17A^+^) cells, and CD8^+^ T cells to the airways at day 8 (Figures 8F–H), although the higher numbers of CD8^+^ T cells did not reach significance. Numbers of recruited NK cells were not different between wild type and knockout animals (Figure 8I). A population of IFNγ positive innate CD19^+^ B cells were transiently recruited to the airways at day 2 post-infection, and this was significantly increased in *Peli1*^−/−^ animals (Figure 8L). Imaging of lung tissues showed inflammatory cell infiltrate (Figure 5), which we characterized using flow cytometry on digested lung tissue after BAL (Figure 9). In the lung tissue of both wild type and knockout animals, influenza A infection resulted in a transient increase in neutrophils and monocytes, similar to that seen in the BALF, as well as an increase in CD4^+^ and CD8^+^ T cells, dendritic cells, and Th17 (CD4^+^IL17A^+^) cells, at 8 days post-infection; again similar to BALF. As observed in the airways, Th17 (CD4^+^IL17A^+^) cells were also significantly increased in the lung tissue of knockout animals (Figure 9G). However, the significant number of neutrophils at day 2 and 4, and CD4^+^ T cells at day 8, of *Peli1*^−/−^ animals in BALF (Figures 8A,F) was not seen in lung tissue (Figures 9A,F), and the significant increase in alveolar macrophages in these animals (Figure 8B) was not observed until day 4 post-infection in lung tissue (Figure 9B). The transient increase of NK cells and CD19^+^ B cells in the airways of knockout and wild type mice (Figures 8I,K) was not observed in lung tissue (Figures 9I,K). Instead, we found an increase in “other macrophages” (CD11b^+^CD64^+^Ly6C^−^; Figure 9D) and γδ T-cells (Figure 9J) in the lung tissue of both strains of mice at day 8.

In summary, our data show that the human lung expresses Pellino-1 and that airway antiviral responses to TLR3 activation and respiratory viruses are regulated by Pellino-1. We show that although Pellino-1 functions as a positive regulator for antiviral responses in isolated epithelial cells and in systemic responses to TLR3 activation, it has the opposite effect in antiviral responses in the complex cellular network of the lung. In *Peli1*^−/−^ mice, TLR3 activation in the lung by synthetic agonists, or by infection with rhinovirus or influenza, is characterized by an increased inflammatory response with excess induction of pro-inflammatory cytokines including IL-6 and TNFα, increased capillary leak, and a modulation of the inflammatory immune response seen as an early increase in neutrophils and innate B cells in the airway and a later increase in T-cell populations in the airway and lung.

## Discussion

The role of ubiquitination in innate immune signaling highlights the therapeutic potential of this pathway to control viral inflammation (Bennett et al., 2012; Moynagh, 2014). Pellino-1 is a potential target since various strands of evidence support its role as a regulator of TLR3 signaling. However, little is known of its specific role in the airway during antiviral responses.

Since Pellino-1 deficiency had been linked to reduced systemic responses to TLR3 agonists, and we had also observed decreased inflammatory responses to respiratory viruses in airway epithelial cells following Pellino-1 knockdown *in vitro* (Bennett et al., 2012), we expected that Pellino-1 deficiency would reduce viral airway inflammation.

We showed that Pellino-1 was expressed in the human lung. Others have shown that Pellino-1 mRNA is increased in COPD (Baines et al., 2011), and although expressed at a protein level in COPD, we did not see differences in expression level between patients and controls. We examined healthy airway epithelial cells from normal donors, and observed that Pellino-1 knockdown reduced responses to TLR3 agonists. We also reconfirmed that *Peli1*^−/−^ mice showed reduced responses to intraperitoneal administration of TLR3 agonists.

To examine the potential for Pellino-1 as a therapeutic target in airways inflammation, we examined its role in murine models of rhinoviral and influenza infection. We studied these two clinically important viruses, as a limitation of mouse models is the poor replication of rhinovirus, and influenza A infection is well-established and characterized. We found that *Peli1*^−/−^ mice did not show the anticipated reduction in the inflammatory responses to the intranasal administration of poly(I:C), nor to rhinovirus or influenza A. In contrast, we observed increases in the initial proinflammatory response of the lung to these stimuli, seen particularly in increased production of cytokines such as TNFα, and evidence of capillary leak. Interestingly, companion studies to the one here and performed in our lab also show that lung responses to bacterial insults are increased when Pellino-1 is knocked down (Hughes et al., 2019). In this study there was an increase in the levels of KC in the lung of *Peli1*^−/−^ animals after non-typeable *Haemophilus influenzae* challenge in a mouse model of COPD, which was associated with increased numbers of recruited neutrophils and enhanced bacterial clearance. In contrast however, we observe no differences in the levels of KC between wild type and knockout mice with either poly(I:C), RV1B, or influenza A. Whilst other models have suggested that Pellino-1 can both positively and negatively regulate the production of IFNs (Enesa et al., 2012; Xiao et al., 2015), we have not seen any significant signs of regulation of IFN pathways in human epithelial cells [these and previous data (Bennett et al., 2012)] or in airway viral load in mice (these data). This suggests that the role of Pellino-1 in the resulting inflammatory response is dependent upon the type of infection.

We studied precision-cut lung slices from mice stimulated *ex vivo* after 48 h in culture, to examine the responses of tissues in the absence of recruited leukocytes. Slices of knockout tissues behaved identically to wild type tissues, suggesting that recruited cells contributed to the phenotype of heightened viral airway inflammation in *Peli1*^−/−^ mice. We did not observe abnormalities in macrophage responses in cells from wild type vs. knockout mice despite data showing Pellino-1 regulates macrophage function (Murphy et al., 2015; Kim et al., 2017). Baseline populations of innate and adaptive immune cells in the airway and lung were similar between wild type and knockout animals, and the recruited cell profile only differed at time points after the initial differences in upregulation of TNFα and IL-6, with increases in pulmonary neutrophil numbers and recruitment of cells such as innate IFNγ-producing B cells, which may enhance responses to viral pathogens in *Peli1*^−/−^ mice, as shown for bacterial pathogens (Bao et al., 2014). Consistent with a potential role for Pellino-1 deficiency to promote excess inflammation, *Peli1*^−/−^ mice are also associated with an increased autoinflammatory activated T cell phenotype later in life (Chang et al., 2011). Pellino-1 deficiency can be associated with increased MyD88 signaling in macrophages (Murphy et al., 2015), and in *Drosophila*, Pellino-1 exerts negative regulation of MyD88 signaling (Ji et al., 2014).

Thus, these data show that Pellino-1 is serving a distinct role in the airway in the context of viral inflammation. Although the roles of Pellino-1 may be more complicated than first appreciated, targeting Pellino-1, or other ubiquitin regulators (Maelfait et al., 2016; Xing et al., 2016), may still prove to be therapeutically useful in viral infection. Our data also show a previously unknown distinct inflammatory signaling pathway in the lung, which will be important if Pellino-1 is targeted therapeutically in the future, in the lung or other systems.

## Data Availability Statement

All datasets presented in this study are included in the article/Supplementary Material.

## Ethics Statement

The studies involving human participants were reviewed and approved by Medical Ethical Committee of the University Medical Center Groninge. The patients/participants provided their written informed consent to participate in this study. The animal study was reviewed and approved by the animal welfare and ethical review bodies at the University of Sheffield and the University of Manchester.

## Author Contributions

EM, EP, HM, LP, IH, WT, MF, TH, MH, JB, S-CS, LB, JS, DD, and IS conceived and designed the experiments. EM, EP, HM, LW, AH, CM, MJ, MF, and BB performed the experiments. EM, EP, MJ, MF, LB, and IS analyzed the data. EM, HM, DD, and IS wrote the paper, with contributions from all authors.

## Conflict of Interest

The authors declare that the research was conducted in the absence of any commercial or financial relationships that could be construed as a potential conflict of interest.
