# Supplementary material for: Pellino-1 Regulates the Responses of the Airway to Viral Infection
Source: Front Cell Infect Microbiol. 2020 Aug 31;10:456. doi: 10.3389/fcimb.2020.00456 (PMC7488214; doi:10.3389/fcimb.2020.00456)
Supplement: Supplementary file 1 [file Data_Sheet_1.PDF]

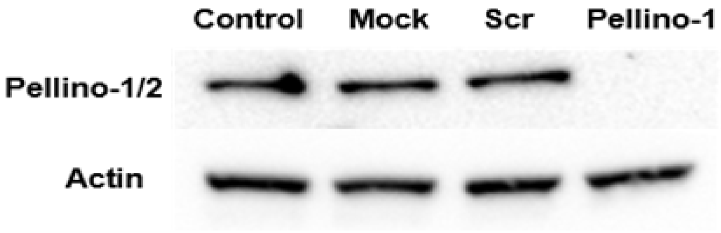

Supplementary Figure S1: Confirmation of Pellino-1 knockdown
Transiently-transfected PBECs were lysed and immunoblotted using antibodies to either Pellino-1/2 (Clone F7, Santa Cruz) or actin (Sigma Aldrich), at 24 h; control cells were untreated; mock cells were stimulated with Lipofectamine 2000 (Invitrogen) only; scrambled (Scr) cells were transfected with 100 nM non-targeting SMARTpool siRNA (Dharmacon); Pellino-1 cells were transfected with 100 nM SMARTpool siRNA targeting Pellino-1 (Dharmacon). Despite using a Pellino-1/2 antibody, we do not find expression of Pellino-2 upon knock down of Pellino-1, supporting our previous findings that Pellino-2 is not expressed in the human airway epithelium (Bennett et al., 2012).
This is a representative blot; knock down was confirmed at each experiment.
