# Supplementary material for: Pellino-1 Regulates the Responses of the Airway to Viral Infection
Source: Front Cell Infect Microbiol. 2020 Aug 31;10:456. doi: 10.3389/fcimb.2020.00456 (PMC7488214; doi:10.3389/fcimb.2020.00456)
Supplement: Supplementary file 2 [file Data_Sheet_2.PDF]

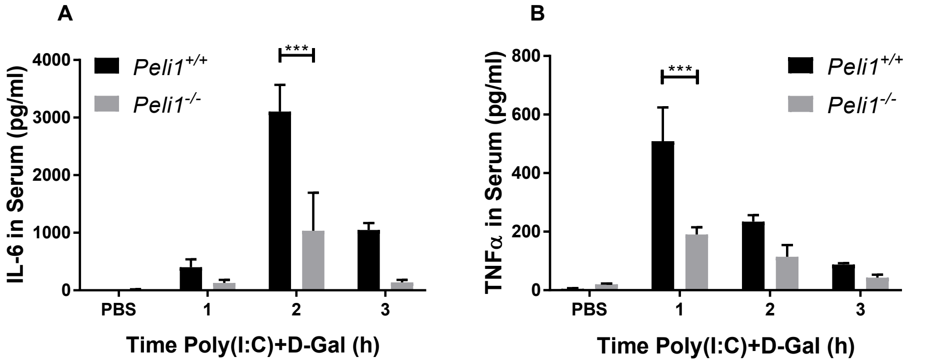

Supplementary Figure S2: Peli1 is required for the proinflammatory response to systemic TLR3 activation

Peli1$^{-/-}$ mice and age- and sex-matched wild type littermate controls were given a single dose of poly(I:C) (0.5 µg/g body weight) plus D-Galactosamine (0.7 mg/g body weight) or PBS (control) i.p. Animals were sacrificed and blood harvested at the time points indicated. Cytokine IL-6 (A) and TNFα (B) levels in venous blood were determined by CBA. Data shown are mean ± SEM; *Peli1*$^{+/+}$ mice PBS n=3, poly(I:C) 1 h n=3, poly(I:C) 2 h n=3, poly(I:C) 3 h n=3; *Peli1*$^{-/-}$ mice PBS n=3, poly(I:C) 1 h n=3, poly(I:C) 2 h n=3, poly(I:C) 3 h n=3. Significant differences between groups are indicated by *** $p<0.001$, as measured by two-way ANOVA with Sidak's post-test.
