# Supplementary material for: Pellino-1 Regulates the Responses of the Airway to Viral Infection
Source: Front Cell Infect Microbiol. 2020 Aug 31;10:456. doi: 10.3389/fcimb.2020.00456 (PMC7488214; doi:10.3389/fcimb.2020.00456)
Supplement: Supplementary file 3 [file Data_Sheet_3.PDF]

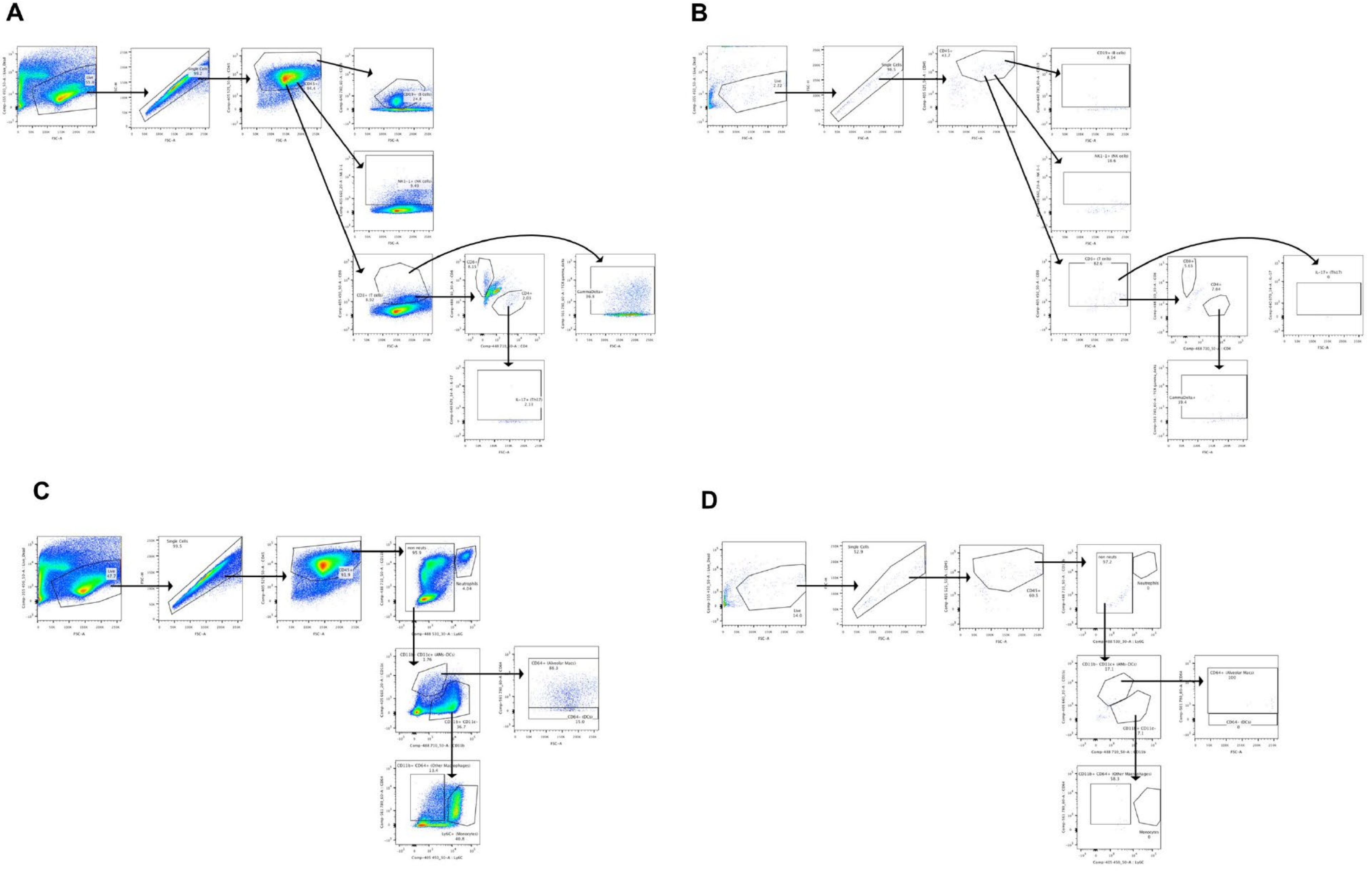

Supplementary Figure S3: Gating strategies for flow analysis

Lymphocyte panel, Influenza A X31 challenged animal (A); lymphocyte panel, PBS challenged animal (B); myeloid panel, Influenza A X31 challenged animal (C); myeloid panel, PBS challenged animal (D).
