# Supplementary material for: Pellino-1 Regulates the Responses of the Airway to Viral Infection
Source: Front Cell Infect Microbiol. 2020 Aug 31;10:456. doi: 10.3389/fcimb.2020.00456 (PMC7488214; doi:10.3389/fcimb.2020.00456)
Supplement: Supplementary file 4 [file Table_1.docx]

**Table 1: Patient characteristics**

| Group | Pellino Staining Ratio (strong/weak) | Age | Gender | COPD Gold Status | FEV1^1^  (% pred) | FEV1/FVC^2^ | Pack Years |
| --- | --- | --- | --- | --- | --- | --- | --- |
| Non-smoker | 0.087997996 | 67 | F | 0 | 114.4 | 80.3 | 0 |
| Non-smoker | 0.164538787 | 63 | F | 0 | Unknown | 84.0 | 0 |
| Non-smoker | 0.222241213 | 50 | M | 0 | 94 | 78 | 0 |
| Non-smoker | 0.036609048 | 70 | F | 0 | 84.5 | 80.9 | 0 |
| Non-smoker | 0.113445654 | 58 | F | 0 | 110.2 | 86.2 | 0 |
| Non-smoker | 0.399540704 | 81 | F | 0 | 116 | 73 | 0 |
| Non-smoker | 0.56879393 | 46 | M | 0 | 96.9 | 81.7 | 0 |
| Non-smoker | 0.019628522 | 70 | F | 0 | Unknown | Unknown | Unknown |
| Current Smoker | 0.014334041 | 57 | F | 0 | 102.9 | 78.4 | 35 |
| Current Smoker | 0.048611491 | 61 | M | 0 | Unknown | 80.0 | 75 |
| Current Smoker | 0.019528669 | 47 | F | 0 | 92.4 | 73.9 | 30 |
| Current Smoker | 0.827603802 | 43 | M | 0 | 94 | 69 | 28 |
| Current Smoker | 0.07487487 | 49 | F | 0 | 90.7 | 82.3 | 33 |
| Current Smoker | 0.040940439 | 54 | M | 0 | 103.8 | 90.1 | 38 |
| Current Smoker | 0.154711379 | 61 | M | 0 | 74 | 80.9 | 14 |
| Current Smoker | 6.251773127 | 42 | M | 0 | 108 | 73 | 8 |
| COPD-Current Smoker | 1.359083498 | 64 | M | 2 | 89 | 64.55 | 80 |
| COPD-Current Smoker | 0.006814649 | 51 | M | 2 | 66.3 | 61.1 | 28 |
| COPD-Current Smoker | 0.058577455 | 70 | M | 2 | 55.5 | 55.2 | 35 |
| COPD-Current Smoker | 0.029763464 | 47 | F | 2 | 61.1 | 75 | 50 |
| COPD-Current Smoker | 0.380605984 | 54 | M | 2 | 74.3 | 65.4 | 14.2 |
| COPD-Current Smoker | 0.239195334 | 61 | F | 2 | 79.7 | 65.1 | 26 |
| COPD-Current Smoker | 0.460247896 | 56 | M | 2 | 67.6 | 59.4 | 30 |

^1^Forced expiratory volume 1 second

^2^Forced expiratory volume/forced vital capacity ratio
